# Sampling challenges in a study examining refugee resettlement

**DOI:** 10.1186/1472-698X-11-2

**Published:** 2011-03-15

**Authors:** Cheryl MR Sulaiman-Hill, Sandra C Thompson

**Affiliations:** 1 Centre for International Health, Curtin University, GPO Box U1987, Perth, Western Australia, Australia; 2 Winthrop Professor, Chair of Rural Health (University of Western Australia) Director, Combined Universities Centre for Rural Health, P.O. Box 109, Geraldton 6531, Western Australia, Australia

## Abstract

**Background:**

As almost half of all refugees currently under United Nations protection are from Afghanistan or Iraq and significant numbers have already been resettled outside the region of origin, it is likely that future research will examine their resettlement needs. A number of methodological challenges confront researchers working with culturally and linguistically diverse groups; however, few detailed articles are available to inform other studies. The aim of this paper is to outline challenges with sampling and recruitment of socially invisible refugee groups, describing the method adopted for a mixed methods exploratory study assessing mental health, subjective wellbeing and resettlement perspectives of Afghan and Kurdish refugees living in New Zealand and Australia. Sampling strategies used in previous studies with similar refugee groups were considered before determining the approach to recruitment

**Methods:**

A snowball approach was adopted for the study, with multiple entry points into the communities being used to choose as wide a range of people as possible to provide further contacts and reduce selection bias. Census data was used to assess the representativeness of the sample.

**Results:**

A sample of 193 former refugee participants was recruited in Christchurch (n = 98) and Perth (n = 95), 47% were of Afghan and 53% Kurdish ethnicity. A good gender balance (males 52%, females 48%) was achieved overall, mainly as a result of the sampling method used. Differences in the demographic composition of groups in each location were observed, especially in relation to the length of time spent in a refugee situation and time since arrival, reflecting variations in national humanitarian quota intakes. Although some measures were problematic, Census data comparison to assess reasonable representativeness of the study sample was generally reassuring.

**Conclusions:**

Snowball sampling, with multiple initiation points to reduce selection bias, was necessary to locate and identify participants, provide reassurance and break down barriers. Personal contact was critical for both recruitment and data quality, and highlighted the importance of interviewer cultural sensitivity. Cross-national comparative studies, particularly relating to refugee resettlement within different policy environments, also need to take into consideration the differing pre-migration experiences and time since arrival of refugee groups, as these can add additional layers of complexity to study design and interpretation.

## Background

Globally, the number of people forcibly displaced by conflict is a continuing source of concern, with over 43 million recorded at the end of 2009 [1]. Almost half of all refugees currently under the protection of the United Nations High Commissioner for Refugees (UNHCR) originally came from Afghanistan or Iraq, and although less than 1% will eventually be resettled in new host nations[1], their long term health and settlement prospects are a matter of continuing relevance. Since 2000, Australia alone has accepted over 58,000 Afghan refugees, while more than 5000 have been re-homed in New Zealand (NZ) [2]. Refugees accepted under humanitarian programs, while eligible for state support frequently denied to asylum seekers, are still vulnerable to acculturative stress, so attitudes and policies to immigration of receiving countries potentially play an important role in resettlement outcomes. Given that Australia and NZ have both accepted refugees for many years and have dedicated settlement policies, an exploratory mixed method comparative study of refugee resettlement experiences in a city in each location was proposed. The main purpose of the study was to compare the resettlement of two distinct refugee groups, Afghans and Kurds, to qualitatively describe their resettlement experiences in Christchurch, NZ and Perth, Western Australia (WA) and provide a quantitative assessment of their health and quality of life, conceptualised as subjective quality of life, psychological well being, and general perceived self efficacy. To achieve this we needed practical strategies for accessing four separate communities and establishing relationships with adult former refugees who had arrived between 1988 and 2008. The aim of this paper is to discuss some of the sampling challenges encountered and describe our approach to study design and practical fieldwork strategies.

### Review of previous studies

To assess the various sampling techniques adopted by other researchers working with similar refugee groups, we conducted a literature search in 2007 using Google Scholar, ProQuest and PubMed databases. Because of our ethnic focus and desire for cultural sensitivity, we only included studies in western settings involving refugee or asylum seeker participants from our target regions, namely Afghanistan, Iraq, Iran or the Kurdish areas of the Middle East. A number of published articles were identified, reporting a range of different approaches to participant selection and strategies for sampling. Many of these had a qualitative focus and as we were principally interested in quantitative sampling strategies, we carefully reviewed nine relevant articles which undertook quantitative assessment and analysis (Table 1). Of these, a range of different techniques were used: one study did not specify the sampling method [3], two obtained random samples (using national survey data, or based on country of birth) [4,5], one approximated a random sample by using a systematic technique where every 4^th ^person was included, and another attempted a random selection in collaboration with clan leaders [6,7]. However, methodological issues with these approaches can be identified. For example, a systematic technique relies on access to a database of refugees that is of sufficient size to allow an adequate sample size to be drawn, and in practice, this is often not feasible. Some concern can also be expressed about a study where collaboration with clan leaders for participant recruitment may introduce gatekeeper bias, or raise concerns about coercion [8]. Of the remaining studies, in two the investigators approached all members of the study group e.g. all asylum seekers registered with an NGO [9,10], while the other two used convenience sampling methods [11,12]. Our eventual conclusion was that there are multiple challenges in obtaining information from these vulnerable refugee population minorities and that no apparent consensus exists on the most appropriate methodologies for use with these populations.

**Table 1:** Selection of published research studies with Afghan, Kurdish, Iraqi or Iranian migrants, refugees or asylum seekers

Author	Sampling strategy	Outcome variables	Study participants
Ahmad et al [6]	Random in collaboration with clan leaders for names. Mixed strategy with different groups	Post Traumatic stress (PTSD) symptoms in traumatized children	78 Kurdish children in Iraq (45 Anfal survivors & 33 orphans), 66 Kurdish refugee children in Sweden & 67 Swedish children

Gerritsen et al [4]	Random by registered country of birth	General health, PTSD, depression & anxiety	178 refugees & 262 asylum seekers (Iranian, Afghan & Somali) in the Netherlands

Ghazinour, Richter & Eisemann [11]	Convenience - clinic patients and volunteers	Sense of coherence, coping resources & social support	100 Iranian refugees settled in Sweden (50 people in outpatient clinic & 50 volunteers)

Gilgen et al [9]	Convenience - All people presenting to Outpatient/General Practice clinic	Health interview for common health problems	36 Bosnian, 62 Turkish/Kurdish & 48 Swiss internal migrants in Switzerland

Hafshejani [12]	Small group convenience	PTSD & meaning in life	59 Iranian & Afghan males who have experienced war, now in Sydney

Husni et al [3]	Not stated	Satisfaction ratings of personal safety, health, employment, food, financial security, social life & entertainment	54 Kurdish refugees, 29 living in the UK & 25 in Canada

Ichikawa, Nakahara & Wakai [10]	All asylum seekers with two Non-Governmental Organisations	Assessment of post-migration detention on mental health	55 Afghan asylum seekers in Japan

Sondergaard, Ekblad & Theorell [7]	Every 4^th ^refugee in group	Life events, ongoing difficulties & self reported health	86 refugees from Iraq (Arabic & Sorani speakers) in Stockholm

Taloyan et al [5]	Random from Swedish National Survey & Level of Living Survey data	Association between ethnicity, poor self reported health, psychological distress, sleeping difficulties & use of psychotropic drugs	Immigrant Kurdish men (n = 111) & native Swedish men (n = 1412) living in Sweden

## Methods

### Study design and instruments

The exploratory study adopted a mixed methods approach, combining qualitative interview responses to open ended questions about health and resettlement experiences with quantitative assessment of psychological distress, subjective wellbeing and general perceived self efficacy. The initial phase of the study involved extensive profiling of each host community through interviews with refugee community leaders, interpreters and cross cultural workers, refugee agencies, health workers, refugee legal advocates and immigration personnel [13-15]. Data obtained guided language considerations and awareness of specific cultural requirements, as well as helping to define the social structure of each community [15]. Pre-translated and culturally validated Farsi, English and Arabic versions of the Kessler-10 Psychological Distress Scale (K-10) [16,17], General Perceived Self Efficacy Scale (GPSE) [18-21], and Australian Unity Well Being Index (Personal Well Being Index PWI) [22] were provided for self completion, or with interpreter assistance if necessary, to quantitatively measure the variables of interest. Nearly 90% of questionnaires were self completed during the first part of the interview, although this varied amongst the groups with almost no Australian Kurds or New Zealand Afghans requiring extra assistance. The second part of the 1-2 hour interview involved more in-depth discussion around open ended questions; this was normally conducted in English. Translations of all additional study materials (information sheets and consent forms), prepared using standard back translation methods [23] were provided in Farsi and Sorani (Kurdish dialect), as well as English, to ensure ethical standards were maintained and to help overcome reticence to participate due to host language limitations [14]. A full description of the reasons for selection and characteristics of the instruments is available elsewhere [24].

Ethical approval for the study was obtained from the Human Research Ethics Committee, Curtin University in Perth, WA.

### Participants

Study participants were of Kurdish or Afghan ethnicity, 18 years or older at the time of the study, who arrived in Australia or New Zealand as refugees or asylum seekers between 1988 and 2008, and who were resident in either Christchurch or Perth at the time of data collection in 2008. The inclusion of participants settled for up to 20 years allowed a longer term focus, and also meant that the Kurdish group resident in Perth, who mostly arrived during the 1990 s, could be included. Although this meant a small number of eligible participants were children at the time of arrival, in practice all except two were of school age and had clear recollections of the resettlement experience. We are unaware of any other studies looking at former refugees up to 20 years post-resettlement, so our findings should be of wider significance.

### Sampling strategy

As one aim of our study was to compare groups, quantitative data was needed, so we wanted to obtain as representative a sample as possible, which reflected the composition of the groups, given these constraints. We acknowledge that truly representative samples can only be obtained through random sampling techniques, but within the context of our exploratory study of previously unstudied groups, our goal was primarily to provide as wide a cross section of participants as possible. Because former refugee communities may be socially invisible and wary of outsiders [13,25], a snowball sampling method, where identification of potential participants, introductions and approval of study objectives and researchers is assured through personal endorsement, was the preferred approach. It has been acknowledged that when 'attempting to study hidden populations for whom adequate lists and consequently sampling frames are not readily available, snowball sampling methodologies may be the only feasible methods available.'[26] However, to reduce selection bias inherent in this method, multiple entry points into the communities were used, choosing as wide a range of people as possible to provide further contacts [13,25,27]. It has been suggested that greater heterogeneity in snowball samples, and improved representativeness can be achieved by increasing sample sizes, using quotas for key demographic variables, use of multiple starting points for snowball initiation and using a small number of links within each chain [13,27]. Similarly, less reliance on community based or gatekeeper organisations for locating potential participants may ensure more marginalised or less socially active individuals are located [13]. With the exception of the Christchurch Kurdish community, between six and eight discrete snowball initiation points were used with each of the other three groups, with a variety of people radiating out from each 'entry' point. Although a quota was not formally applied, care was taken to ensure that these key people represented a cross section of each community by including a range of different Afghan ethnic groups (Hazara, Tajik, Uzbek etc) and religious affiliations (Sunni, Shi'a etc) who move in different social circles [8]. Contacts were not generally sourced through refugee resettlement agencies to reduce possible gatekeeper bias [13,27] in selection of individuals who could provide 'appropriate' responses and also to minimise identification of the study with agency agendas. For the New Zealand Kurdish group however, the small population size and cohesive, well structured community meant all eligible adults could be approached. As our study was exploratory, rather than hypothesis testing, sample size was based upon logistical considerations, including the small size of the communities from which respondents could be drawn, so our aim was to recruit between 40-50 individuals from each group to provide sufficient data for some basic descriptive statistical comparison. The specific strategies used for community access in each location are described in Table 2.

**Table 2:** Sources of Community Access - Kurdish Afghan Refugees NZ and Australia study (KARNZA)

New Zealand	
Limited access to the communities was already established through personal and professional connections with Refugee Services Aotearoa, refugee and Muslim groups in Christchurch.	
***Kurdish*** (Estimated community size 180-200)	The small size and cohesion of the group enabled all adult community members to be contacted and former refugees invited to participate. Initial introduction to families was through the Kurdish committee chairperson & interpreters, who provided follow up contact details. Religious (Eid), ethnic (Newroz) & marriage celebrations were attended by the lead researcher and proved valuable for establishing relationships. **Total number recruited**: 49 (27 male (55%), 22 female (45%))
***Afghan*** (Estimated community size 1000-1200)	Initial contact points included community leaders from Afghan & Refugee associations, cross-cultural workers & interpreters, Afghan sports teams and existing contacts. A total of eight contacts provided discrete links for further referrals. **Total number recruited**: 49 (32 male (65%), 17 female (35%))

**Australia**	

No prior connections with either group in Perth necessitated a more general approach. Websites for ethnic and Muslim groups provided initial links and phone numbers, also Muslim women's support groups and ESOL language classes. Afghan & Kurdish academics at Curtin university provided background data on community profiles and contacts.	
***Kurdish*** (Estimated community size 1000-1500)	Initial contacts were with members of the Kurdish committee responsible for organising a Newroz festival event, which was subsequently attended by the lead researcher. They provided additional contact information for interpreters who assisted with data collection. Several meetings took place in Kurdish-owned kebab restaurants, popular spots for community members to congregate. Additional contacts were also obtained from independent Muslim sources. A total of six separate contact people provided snowball initiation points. **Total number recruited**: 54 (28 male (52%), 26 female (48%))
***Afghan*** (Estimated community size 1000-1500)	Six discrete people were also used to initiate sampling in the Afghan group. These included community leaders for different ethnic groups & Afghan associations, Muslim women's organisations and cross-cultural workers. ESOL classes with Farsi-speaking interpreters also proved useful for recruitment of a mixture of Afghan women. **Total number recruited**: 41 (13 male (32%), 28 female (68%))

*Note*: Estimates of community size were provided by representatives of the respective groups

### Statistical analysis of sample characteristics

Quantitative data was analysed using SPSS 12.0 (SPSS Inc.). Frequency distributions for demographic variables were calculated, with results split by resettlement location and refugee community. Non-parametric tests were performed to assess differences between groups.

## Results

The sampling response rate for each group varied. For the Kurds in Christchurch 65% of eligible people who were approached participated, while snowballing within the Christchurch Afghan community achieved an 85% response. For both Kurdish and Afghani groups in Perth the response rate was only about 40%, even with snowball endorsement.

The overall sample of 193 participants (Christchurch n = 98, Perth n = 95) reflected a good spread of participants, with a range of opinions and experiences (Table 3). There was a good gender balance, age distribution, family size (1-10), time spent as a refugee (< 1-27 years), and time since resettlement (< 1-20 years). Education level was determined by the number of years of schooling with less than one year coded as none/minimal, 1-6 years as primary, 7-13 years as secondary, and the remainder classified as tertiary level.

**Table 3:** Summary of participant demographics

Participant characteristics	Number of participants (n = 193)						
		
	Total (%)	Afghan	Kurdish	Chch	Perth	Statistic	*p*
***Refugee community***							
Afghan	90 (47)	90		49	41	χ2(1,193) = 0.91 (location*)	0.341
Kurdish	103 (53)		103	49	54		
***Gender***							
Male	100 (52)	45	55	59	41	χ2(1,193) = 0.22 (community**)	0.637
Female	93 (48)	45	48	39	54	χ2(1,193) = 5.6 (location)	0.018
***Age***							
18-19 years	17 (9)	4	13	11	6		
20-29 years	62 (32)	35	27	29	33		
30-39 years	56 (29)	25	31	26	30		
40-49 years	33 (17)	11	22	19	14		
50-59 years	17 (9)	10	7	8	9		
60 years & over	8 (4)	5	3	5	3		
***Marital status***							
Married	125 (65)	59	66	59	66	χ2(1,191) = 2.51 (community)	0.474
Never married	55 (28)	24	31	31	24	χ2(1,191) = 2.06 (location)	0.561
Previously married	11 (6)	5	6	7	4		
***Religion***							
Muslim	180 (93)	88	92	88	92		
Non-Muslim	8 (4)	1	7	7	1		
***Education***							
None	14 (7)	5	9	9	5	χ2(1,193) = 10.26(community)	0.017
Primary	28 (15)	9	19	25	3	χ2(1,193) = 22.70 (location)	0.000
Secondary	100 (52)	43	57	45	55		
Vocational/university	51 (26)	33	18	19	32		
***Continuous Variables***	***Range***						
Time as refugee yrs (Median)	0-27 years	4.0	8.0	15.0	2.0	U = 3451.5, Z = -2.74 (community)	0.006
						U = 2159.0, Z = -6.24 (location)	0.000
Time since resettlement yrs (Median)	0.5-20 years	6.0	7.0	4.0	11.0	U = 3675.0, Z = -2.38 (community)	0.017
						U = 1459.5, Z = -8.22 (location)	0.000
Mean household size (SD)	1-10 people	5.0 (1.8)	5.4 (2.0)	5.5 (1.9)	5.0 (1.9)		

*Note*: Some totals do not sum to 193, missing data not included

*Location - Christchurch (Chch) & Perth

**Community - Afghan & Kurdish

No significant differences in participant numbers between groups were noted for the total number of Afghan and Kurdish participants, the total number of participants in Christchurch and Perth, marital status, and household size. However, significant gender differences by location were found, with more males in Christchurch (60%) than Perth (43%). Differences by resettlement location and between refugee communities were also observed for the total time spent in a refugee situation (Kurds longer than Afghans, and those in Christchurch longer than people in Perth), the time since resettlement (participants in Perth settled longer), and education level of participants (those in Perth reported more years of schooling overall than the group in Christchurch).

### Representativeness of the sample

Lack of a clear sampling frame [13,25,26], a widely acknowledged problem with refugee research, and limitations with Census data [8,13,28] hinder attempts to accurately gauge representativeness. However, to assess the likely representativeness of our sample, aggregated Afghan and Kurdish data was compared with 2006 Census data for Western Australian (WA) residents born in Afghanistan, Iran and Iraq (Table 4) [29-31]. Appropriate Census data from New Zealand was not available for comparison, although anecdotal reports from within the communities suggest that our sample reflected local demographics. With such hidden populations, it has been suggested that construction of a 'tentative map' of community demographics developed in consultation with professionals working with the target groups or community members may be helpful in order to judge representativeness [26]. In our case, this was done informally through consultation and discussion with community members. Due to the invisibility of Kurdish ethnicity in immigration statistics, Iraqi and Iranian census data was used as a compromise substitute, although significant limitations exist with this. In particular, the Kurdish geographic region spreads across Iran, Iraq, Turkey, and Syria, with small populations elsewhere in the Middle East, and it is an individual's country of birth that is recorded in government statistics. As the Kurds comprise minority groups in these countries, a direct comparison with natal population demographics is likely to be misleading. However, as shown in Table 4 our sample did achieve a good gender and age balance when compared with the ethnic populations in WA. Religion was predominantly Muslim in our sample, which is consistent for people of Afghan and Kurdish background. The Iran and Iraq-born groups in WA also include a significant number of Baha'is, Armenian apostolic adherents and Catholics who sought refuge from religious persecution as minorities in the 1980 s [31], which explains the disparity with this variable. Overall, our Afghan sample was slightly better educated (59% with some form of tertiary qualification, compared with 34% of Afghans in WA), and both groups were settled longer (almost half arrived before 1996, compared with about one third of the respective populations). Our sample also appears to speak better English; however, this result is distorted by the definition of speaking English. In the Census, participants are asked to rate their English ability as speaking English very well, well, not well or not at all; only those who speak it well or very well were included in the table data. In comparison, we dichotomised responses into functional/no functional English to assess levels of English literacy, so our results will appear positively skewed for this variable.

**Table 4:** Comparison of total sample demographics with 2006 Census data for Western Australia

		Afghan sample (n = 90)	Census Afghan born (n = 1460)	Kurdish sample (n = 103)	Census Iraq born (n = 1680)	Census - Iran born (n = 2190)
**Place of birth**	Iran	4		39		
	Iraq			59		
	Afghanistan	78				
	India	4				
	Pakistan	3				
	Turkey			3		
	Not stated	1		2		
**Gender**	Male	50%	54%	53%	53%	52%
	Female	50%	46%	47%	47%	48%
**Median age**		20-29	28.9 years	30-39	35.7 yrs	40.4 yrs
Religion*	Muslim	98%	95%	89%	31%	32%
Speaks English **		76%	68%	95%	71%	81%
**Post school** qualifications ***		59%	34%	33%	33%	59%
**Arrival pre-1996**		46%	27%	52%	33%	55%

*Note that census data for Iraq and Iran-born people includes groups other than Kurds. The Iraq and Iran-born groups include Bahai's and Christians, many of whom sought refuge from religious persecution as minorities in these countries. In comparison, the majority of Kurds are Muslim with small numbers following traditional Kurdish religions. These differences are not reflected in census data.

**Refugee study data includes people with varying English language skills. They were not asked to rate their English ability, just whether they could speak functional English or not, so percentages are likely to be higher than census data reporting the ability to speak English well.

*** Census data also includes people aged 15-17 who are still likely to be at school, whereas the study included those 18 and older who have mostly left school with many going on to further study

## Discussion

Numerous methodological and ethical challenges arise when conducting research studies with refugee or migrant groups. One fundamental decision is whether to adopt a quantitative or qualitative approach. Both methodologies have their own merits, depending on the research focus and desired outcomes and can be distinguished primarily by the type of data obtained (text based or numeric), the underlying logic employed (inductive or deductive), method of analysis used (interpretive or statistical) and the presumed underlying paradigm (positivist/rationalistic or interpretive/critical/naturalistic) [32]. Increasingly, researchers are adopting mixed method approaches, employing innovative strategies to combine methods that attempt to both generalise results to a wider population, while also generating in-depth understanding of individual cases [32,33]. Mixed method techniques can be used to expand the scope of a study, to corroborate data, provide deeper insights, or aid ongoing development of a project. In our study, quantitative instrument-based data was considered to provide complementary data (rather than for statistical inference) to corroborate interview findings and allow idiographic generalisations about study participants rather than nomothetic generalisation about the populations as a whole [33]. Kessler-10 scores, for example, could also be used for qualitative profiling, by allowing further in-depth qualitative analysis for participants scoring within the high or very high risk of psychological distress range.

One of the main methodological concerns with mixing methods arises when selecting the type of sampling strategy to be used. Probability techniques, such as simple random, stratified (sampled to meet fixed quotas based on previously defined variables), systematic random or cluster sampling methods underlie quantitative methodologies, allowing statistical inferences based on generalisations from the sample to a wider population to be drawn [33-35]. These are also referred to as descending methodologies because of the focus on the general population from which the sample is drawn. In contrast, purposeful or non-probability sampling is an ascending method, working up from individual cases to draw conclusions and generate idiographic knowledge [33]. Common techniques include convenience sampling, where cases are chosen because they are readily identified or available, quota sampling (similar to stratified sampling but cases are not randomly selected), random purposeful (selection from large pool of info-rich cases) or stratified purposeful methods (based on a pre-set quota of info-rich cases) and snowball sampling (mainly used to identify rare cases, where sampling depends on referrals from existing cases to generate potential participants) [33,35]. For many studies the design and sampling technique will be dictated by individual research goals, however the unique set of challenges encountered when working with hidden or hard to reach populations, such as refugee groups, necessitates a more pragmatic approach. In practice, most research studies with forced migrants employ some form of non-probability sampling, adopt an ethnographic community participatory approach [8,14], or in some cases are able to utilize pre-existing data sources, such as Census or Immigration statistics [13,28] although these have only limited capacity for analysis of specific issues or identification of former refugees.

A 'hidden population' refers 'to a subset of the general population whose membership is not readily distinguished or enumerated based on existing knowledge and/or sampling capabilities' [15]. The lack of clear sampling frames means that snowball sampling may be the only feasible way of locating potential participants [26,27], despite concerns around selection bias [13,25-27]. It is conceivable that sampling frames may exist for some sub-groups of refugees, for example those currently within a resettlement program [13], however for those settled longer, individuals who may have internally migrated, asylum seekers or families arriving under reunification programs, few, if any, records will be available. Many minority groups may be socially invisible, effectively hidden within existing population statistics, which is a particular issue for those of Kurdish ethnicity who are normally categorised by their country of birth (mostly Iraq, Iran, Syria or Turkey), and presents a particular challenge for recruitment. It is not possible to obtain accurate immigration or census data for these groups, so utilisation of nationality-based databases for random selection is not feasible.

Sampling challenges are of fundamental concern when the entire validity of a research study may be questioned depending on the method selected. Due to sampling difficulties, refugee-focussed research projects often either utilise existing epidemiological databases, are conducted on a large scale through governmental or health organisations where attempts at randomisation with multiple ethnic groups are feasible, or employ purposive sampling on a smaller scale with a qualitative focus. This presents a dilemma when attempting to obtain comparable data between groups or locations; either some compromise is required to access and recruit participants, or ethnic minority groups risk exclusion from comparative research. Alternatively, studies may rely solely on qualitative content, which although offering valuable insight into individual experiences, only tells part of the story and potentially limits monitoring of resettlement outcomes. For this reason, a mixed methods approach, incorporating both qualitative and quantitative dimensions was our preferred option, although as identified during our review of previous studies, there are significant limitations with obtaining representative refugee samples with groups from our region of interest for quantitative studies. For us, and most people with an interest in the health of refugee populations, there is need reach a pragmatic compromise between representativeness and logistic feasibility; without this, there would be a lack of evidence on the health and needs of such vulnerable populations.

Both Christchurch and Perth provide dedicated settlement services and targeted support for refugees, but accurate demographic data of specific communities is scant. Obtaining statistically representative samples of such socially invisible groups is known to be problematic [13,25], given the limited size of communities, their invisibility in national data sets, the target participants' concern about research motives, power differentials between participants and the researchers, as well as difficulties with access and trust [26,36]. For refugees, some reticence may also be related to pre-migration experiences, so extra care and sensitivity around the establishment of relationships is necessary. When four distinct communities are involved, these issues are multiplied. It was important to develop good relationships with leaders and high profile members of each community, to build rapport, establish our credentials, discuss research objectives and plan access strategies [14]. This initial preparatory phase, during which we also needed to address language concerns and instrument selection, had an inevitable impact on the time required for the research. The method described here utilised a combination of strategies; the entire Kurdish group in Christchurch was contacted, as it was small and well defined, however the size and difficulties with access to the other three groups necessitated a different approach. By employing a snowball method, we were able to access socially invisible individuals and provide reassurance about our objectives, while at the same time attempting to obtain a cross-sectional representation of the population by using multiple contacts from different groups and backgrounds. The sample size was deliberately large to enhance heterogeneity, and we had multiple, small snowball chains with initial selection guided by an informal quota [13].

Although limitations in the generalisability of this approach are acknowledged, one strength of this method was in the number of women recruited; especially those with limited education who are sometimes overlooked in research studies and would often decline to participate if approached directly. Overall we achieved a good gender balance (male 52%, female 48%), although when broken down by location the proportion varied amongst Afghan participants, with females being 35% in Christchurch, compared with 68% in Perth. Some variation may be explained by our initial recruitment strategies; as one Christchurch entry point was a male Afghan soccer team whose female relatives were generally not interested, while in Perth we were able to directly approach women through support groups and ESOL classes, achieving a good response rate. Differences in social demographics may also play a part, with many Afghan men who had settled longer in Australia too busy working to participate. In comparison, women who were often at home or attending classes during the day welcomed the opportunity to express their views as it provided them a sense of purpose and empowerment [37]. In many cases, the men initially arrived as asylum seekers, subsequently being joined by family members under reunification programs; this accounted for some gender disparity in settlement time as well as English language ability. We found more variation among different Afghan ethnic groups in Christchurch, with gender balance among Hazaras but fewer women of other ethnicities chose to participate. Interestingly, recently arrived women in Christchurch (1-2 years resettled) were more motivated to participate than those settled longer in both locations.

The groups in Christchurch had all arrived within the previous ten years, whereas over 40% of the Afghans and 60% of Kurds in Perth had been settled between 11-20 years. It was notable that the attitudes and behaviour of many of those resettled longer were more suspicious of the research objectives and less hospitable. Perhaps this was a reaction to Australian society where refugee issues are hotly debated in the media and research on these topics more common than in New Zealand, or maybe they no longer thought of themselves as refugees. Whether it was a result of research fatigue [27], a response to public attitudes towards refugees and migrants, or simple apathy, but the opportunity to visit people in their own homes to establish relationships was not really encouraged in Perth. Many Australian participants preferred minimal contact, to meet in a neutral place, or simply to complete questionnaires and return them by mail. The biggest challenge, particularly in Australia, was generating initial interest to overcome apathy and apparent suspicion, with the key feature being identification of enthusiastic people for snowball initiation. Without the chain referral endorsement of snowball sampling, it is unlikely that sufficient interest would have been generated to recruit sufficiently for a viable study. Bloch also noted differences in attitudes between similar refugees in the United Kingdom and South Africa, suggesting that larger numbers of refugees and asylum seekers and more political activists in the UK lead to increasingly suspicious attitudes to research motives [13]. Our Australian experience was a marked contrast to the support and hospitality received in Christchurch where it was not uncommon for participants to want to entertain with lavish meals once the interview was completed. Although this may be partly explained by the fact that some pre-existing relationships existed, the majority of New Zealand participants were total strangers prior to recruitment. For many, especially more recently arrived women, it was the first time their views and opinions had been sought, so participation proved a novel and rewarding experience which was often fuelled by altruistic motives to help other refugees through their own insight and experience [8,37].

Obtaining access to the different communities was a challenge. Overall, it was easier with the groups in Christchurch because we had a better understanding of community dynamics and already established connections with key individuals. Building personal relationships is essential, and an appreciation of customs and social mores fundamental to establishing researcher credentials and acceptability within the group. Taking the time to drink tea and connect on a personal level prior to commencing the formal part of the interview greatly influenced the quality of data obtained. We must have consumed hundreds of cups of tea during the data collection phase, but the opportunity to interact and chat informally provided valuable insights and enhanced our standing overall. The fact that the interviewers were Muslim also improved our respectability, especially for many older people, and helped overcome barriers despite the language issues. Maintaining Islamic proprieties was also important; ensuring interviewers were of an appropriate gender and having an accompanying male relative escort the lead interviewer when necessary, was appreciated and commented on favourably by many participants.

The selection of groups for cross-country studies poses another dilemma, as evidenced by the demographic balance of our sample. Although the entire NZ sample arrived during the previous 10 years, more than half the Australian group were settled longer than this time, in some cases up to twenty years, and this could potentially limit the relevance of some comparative analyses. Variations in migration patterns and conflict situations, national resettlement quotas determined annually in consultation with UNHCR requirements, the presence of existing communities, and many other considerations, result in different refugee groups being accepted for resettlement at different times in different countries. The question arises whether it is better to compare groups based on temporal considerations or ethnic similarity. Because the majority of Afghans and Kurds are Muslims, many are from traditional backgrounds and include members who suffered ongoing ethnic and religious persecution as minority groups; we considered comparison between locations required that the study groups be ethnically similar.

## Conclusions

Research undertaken with refugee groups presents a unique set of challenges. The adoption of a mixed methods approach allowed us to obtain a comparative evaluation of participants' health and well being status, while also providing qualitative feedback on their resettlement experiences. Although the choice of sampling strategies presented a significant dilemma, an ascending methodology was necessary to locate and identify potential participants, as well as providing reassurance about our motives and helping to break down barriers. Using a qualitative interview approach, taking time to engage with participants in an unhurried fashion, was also critical to our success and appreciated by many interviewees who complained that people they have contact with outside their own community are often too rushed or formal to accept their hospitality. The combination of data obtained allows meaningful comparison between groups to be made and conclusions drawn based on both quantitative findings and an in-depth understanding of their perspectives and concerns. In particular, qualitative feedback provided critical information about ongoing sources of stress, as well as negative and positive influences on their quality of life, contextualising the K-10 and PWI findings and suggesting strengths and weaknesses in existing resettlement support programs as well as highlighting potential areas for future research.

Although an increase in the number of refugees has been predicted, there is at present a paucity of detailed methodological articles available for researchers developing study protocols for use with ethnic minority groups. The major limitations of our approach concern a lack of representativeness if care was not taken to adequately survey the target communities in order to guide recruitment and snowball initiation points, and generalisability to a wider population if statistical inference and hypothesis testing was desired. However, despite these concerns, our experience, with its description of limitations and considerations may help inform other studies.

## Competing interests

The authors declare that they have no competing interests.

## Authors' contributions

CS-H conceived the study, participated in its design, co-ordination and data collection, and drafted the manuscript. ST participated in the design of the study and helped draft the manuscript. Both authors read and approved the final manuscript.

## Pre-publication history

The pre-publication history for this paper can be accessed here:

http://www.biomedcentral.com/1472-698X/11/2/prepub
